# Inflammatory fibroid polyp of the ileum presenting with small bowel obstruction in an adult patient: a case report

**DOI:** 10.1186/1752-1947-4-291

**Published:** 2010-08-30

**Authors:** Toygar Toydemir

**Affiliations:** 1 Department of General Surgery, İstanbul Surgery Hospital, Ferah sokak no:18 nişantaşı, 34365 İstanbul/Turkey

## Abstract

**Introduction:**

Inflammatory fibroid polyps are rare benign tumors of the gastrointestinal tract with the gastric antrum being the most common site, followed by the ileum. Histogenesis is still unknown and controversial. Inflammatory fibroid polyps are one of the rare benign conditions leading to intestinal obstruction in adults.

**Case presentation:**

A 54-year-old Caucasian man presented with acute abdomen pain and a two month history of intermittent cramping and lower abdominal pain. Computed tomography imaging demonstrated a partial intestinal obstruction in the location of the terminal ileum. An ileo-ileal intussusception due to a mass lesion 15 cm proximal to the caecum was found on exploratory laparotomy. Intussusception was spontaneously reduced during exploration and a wedge resection was performed to the affected bowel segment. Histopathologic examination showed the mass to be an inflammatory fibroid polyp.

**Conclusion:**

Although inflammatory fibroid polyps are rare and benign, in the case of intestinal obstruction the only solution is a surgical approach.

## Introduction

Intussusception is an uncommon cause of intestinal obstruction in adults [1]. Patients with intussusception present with either acute or chronic intermittent symptoms. The majority of adult intussusceptions occur due to malignant processes [2]. We report the case of an adult inflammatory fibroid polyp (IFP) confined to the terminal ileum which presented with acute symptoms and a repeated intussusception background history. The aim of this study is to remind that some very rare etiologies may be involved in adult intestinal obstructions.

## Case presentation

A 54-year-old Caucasian, Turkish man presented to the emergency department with acute abdominal pain, nausea and vomiting and a two month history of intermittent lower abdominal pain. There was no history of previous abdominal surgery, smoking or alcohol consumption.

On examination, he was uncomfortable and had a heart rate of 110, blood pressure 140/70 mmHg, and a temperature of 37.5°C. Generalized abdominal pain was found on abdominal examination without signs of peritoneal irritation. Bowel sounds were normal. Laboratory analysis revealed 15,000 leukocytes with a prevalence of neutrophils. Other parameters were within the normal limits. Abdominal radiology demonstrated a few air-fluid levels in the right lower quadrant. An intravenous and oral contrast computed tomography showed partial intestinal obstruction in the terminal ileum without generalized small bowel dilatation.

**Figure 1:**
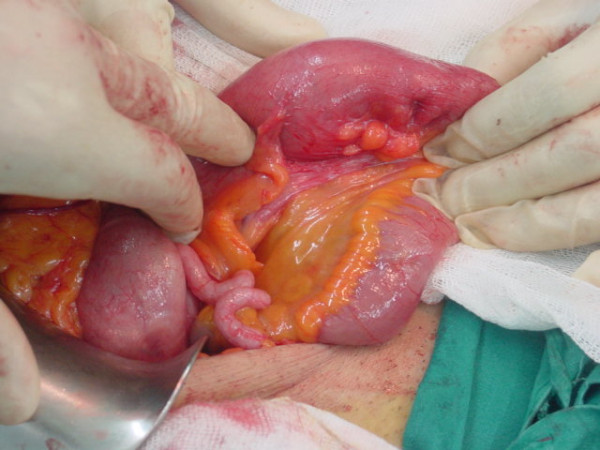
**The lead point of intussusception**.

**Figure 2:**
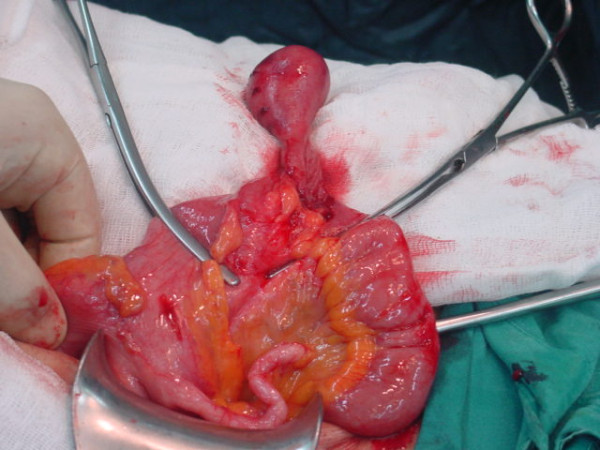
**The view of IFP before resection**.

An exploratory laparotomy was performed with the diagnosis of subacute intestinal obstruction. An intussusception with a mass lesion at its lead point approximately 15 cm proximal to the caecum was found. Intussusception was spontaneously reduced during exploration. Limited edema at the lead point of the bowel, was the only sign of the intussusception (Figures 1 and 2). A wedge resection was performed to the affected bowel segment.

Histopatologic examination showed proliferated vessel formations in a mixoid stroma and inflammatory cell infiltration consistent with IFP.

## Discussion

IFPs are among the least common benign lesions of the gastrointestinal tract. They originate from the submucosa as a solitary or sessile lesion with an inflammatory basis. They can occur throughout the intestinal tract but most frequently in the gastric antrum and small bowel [3]. IFPs usually measure between two and 5 cm in diameter. However, there are also giant IFPs with a size of up to 12.5 cm in diameter having been reported [4]. IFP was first described by Vanek as a 'gastric submucosal granuloma with eosinophilic infiltration' in 1949 [5]. Histologically, IFPs are characterized by vascular and fibroblast proliferation with an eosinophilic inflammatory response. The underlying cause of IFP remains uncertain. Many factors have been suggested as a trigger such as intestinal trauma or eosinophilic gastroenteritis.

IFPs are usually asymptomatic, identified during endoscopy or laparotomy. When they are symptomatic the clinical presentation is determined by the anatomic location. Gastric IFPs may lead to pyloric obstruction or anemia with chronic bleeding [6]. When they arise from the small bowel, intussusception is the most common clinical finding.

Adult intussusception is a very rare condition, accounting for 1% of all adult bowel obstruction and occurs in only 5% to 16% of all intussuscepted cases [2]. About 70% to 90% of intussusception cases are due to benign or malignant neoplasms as a lead point and IFPs, lipomas and adenomas are the benign causes of intussusception [7]. However, it has been shown that intussusception can occur without significant pathological cause [8].

Unlike the more common idiopathic intussusception found in children, intussusception in adult patients still remains a surgical disease. The type of surgical procedure depends on the patient's medical history (previous operations, malignancy) and intra-operative findings [9]. The optimal surgical management of intussusception in adult patients is influenced by two major factors: the presence of distinct malignancy and the local factors such as the degree of associated edema, and relative ischemia of the involved bowel. A wedge resection of affected bowel segment was performed in our case as very limited edema was observed at lead point of intussusception. However, attempts at local removal of polyps through a limited enterotomy, or by wedge resection through edematous bowel, may be dangerous and healthy bowel margins must be secure during segmental resection.

## Conclusions

İntussusception is a very rare cause of adult intestinal obstruction and IFP is one of the least common causes of this rare condition. Although IFPs are benign lesions, surgical treatment is the only solution when they present with small bowel obstruction.

## Competing interests

The author declares that they have no competing interests.

## Authors' contributions

TT is the only author of this paper. TT designed the study and wrote the manuscript.

## Consent

Written informed consent was obtained from the patient for publication of this case report and any accompanying images. A copy of the written consent is available for review by the Editor-in-Chief of this journal.
